# Oleic acid and peanut oil high in oleic acid reverse the inhibitory effect of insulin production of the inflammatory cytokine TNF-α both *in vitro *and *in vivo *systems

**DOI:** 10.1186/1476-511X-8-25

**Published:** 2009-06-26

**Authors:** Evros K Vassiliou, Andres Gonzalez, Carlos Garcia, James H Tadros, Goutam Chakraborty, Jeffrey H Toney

**Affiliations:** 1 Department of Biological Sciences, Kean University, 1000 Morris Avenue, Union, New Jersey 07083, USA; 2 Department of Medicine, St. Joseph's Hospital, 703 Main Street, Paterson, New Jersey 07503, USA; 3 Department of Neurochemistry, Nathan Kline Institute, 140 Old Orangeburg Road, Orangeburg, New York 104624, USA; 4 College of Natural, Applied and Health Sciences, Kean University, 1000 Morris Avenue, Union, New Jersey 07083, USA

## Abstract

**Background:**

Chronic inflammation is a key player in pathogenesis. The inflammatory cytokine, tumor necrosis factor-alpha is a well known inflammatory protein, and has been a therapeutic target for the treatment of diseases such as Rheumatoid Arthritis and Crohn's Disease. Obesity is a well known risk factor for developing non-insulin dependent diabetes melitus. Adipose tissue has been shown to produce tumor necrosis factor-alpha, which has the ability to reduce insulin secretion and induce insulin resistance. Based on these observations, we sought to investigate the impact of unsaturated fatty acids such as oleic acid in the presence of TNF-α in terms of insulin production, the molecular mechanisms involved and the in vivo effect of a diet high in oleic acid on a mouse model of type II diabetes, KKA^y^.

**Methods:**

The rat pancreatic beta cell line INS-1 was used as a cell biological model since it exhibits glucose dependent insulin secretion. Insulin production assessment was carried out using enzyme linked immunosorbent assay and cAMP quantification with competitive ELISA. Viability of TNF-α and oleic acid treated cells was evaluated using flow cytometry. PPAR-γ translocation was assessed using a PPRE based ELISA system. In vivo studies were carried out on adult male KKA^y ^mice and glucose levels were measured with a glucometer.

**Results:**

Oleic acid and peanut oil high in oleic acid were able to enhance insulin production in INS-1. TNF-α inhibited insulin production but pre-treatment with oleic acid reversed this inhibitory effect. The viability status of INS-1 cells treated with TNF-α and oleic acid was not affected. Translocation of the peroxisome proliferator- activated receptor transcription factor to the nucleus was elevated in oleic acid treated cells. Finally, type II diabetic mice that were administered a high oleic acid diet derived from peanut oil, had decreased glucose levels compared to animals administered a high fat diet with no oleic acid.

**Conclusion:**

Oleic acid was found to be effective in reversing the inhibitory effect in insulin production of the inflammatory cytokine TNF-α. This finding is consistent with the reported therapeutic characteristics of other monounsaturated and polyunsaturated fatty acids. Furthermore, a diet high in oleic acid, which can be easily achieved through consumption of peanuts and olive oil, can have a beneficial effect in type II diabetes and ultimately reverse the negative effects of inflammatory cytokines observed in obesity and non insulin dependent diabetes mellitus.

## Background

Acute inflammation is utilized by the immune system to effectively isolate and eliminate pathogenic microorganisms. Among the most commonly observed cytokines in inflammatory microenvironments is Tumor Necrosis Factor-alpha (TNF-α) [1]. This cytokine is produced by a variety of immune cells and is pleiotropic in nature[2]. TNF-α is also produced by adipose tissue and it is thought to play a major role in the Metabolic Syndrome (MS), which is characterized by insulin resistance and inflammation[3]. Studies utilizing the INS-1 glucose dependent insulin secreting cell line, have shown that TNF-α is capable of inhibiting insulin secretion[4,5]. Furthermore, TNF-α interferes with insulin signalling in a variety of non-insulin producing cells, essentially inducing a state of insulin resistance[6]. The concept of chronic low level inflammation playing an important role in a number of diseases is beginning to gain acceptance in the medical community. The anti-inflammatory properties of cholesterol lowering drugs, collectively referred to as statins, is an excellent example that provides support for this concept. The anti-inflammatory therapeutic effect of statins on individuals that have normal cholesterol, but have elevated high sensitivity C-reactive protein (hsCRP), an inflammatory marker, was shown in the recently completed Jupiter study[7]. This finding is strongly indicative of the importance of regulating inflammation and ultimately avoiding disease in the future.

Polyunsaturated Fatty Acids (PUFAs) and Monounsaturated Fatty Acids (MUFAs) have received a lot of attention in the last decade and their health benefits are becoming increasingly evident [8-10]. Due to the widespread practice of hydrogenation in processed foods, the levels of saturated fatty acids being consumed by populations residing in developed countries has increased significantly and the subsequent increase in cardiovascular disease and metabolic disorders is becoming a significant public health issue [11]. The specific mechanisms through which unsaturated fatty acids exert their therapeutic properties are quite varied. Metabolites of Eicosapentaenoic Acid (EPA) and Docosahexaenoic Acid (DHA) collectively referred to as Resolvins, have been shown to be potent anti-inflammatory agents[12,13]. A number of unsaturated fatty acids have also been shown to bind the Peroxisome Proliferator-Activated Receptor-gamma (PPAR-γ)[14]. The binding of free fatty acids to PPAR-γ has been shown to result in the translocation of the receptor to the nucleus and regulation of gene expression. Contrary to PUFAs and MUFAs, saturated fatty acids are associated with inflammation at the level of adipose tissue[15]. Mediterranean populations with dietary habits that are high in MUFAs, partly due to high olive oil consumption, have considerably reduced inflammatory markers when compared to populations that have lower oleic acid intake [16-18].

In this study, we show that oleic acid and peanut oil high in oleic acid can reverse the inhibitory effect of the inflammatory cytokine TNF-α on insulin production both *in vitro *and *in vivo*. Furthermore, a peanut oil diet high in oleic acid can reverse hyperglycemia in a mouse model of type II diabetes. These findings further support the use of diet manipulation to treat diseases that have an inflammatory aetiology.

## Methods

### Cell cultures

The glucose sensitive rat pancreatic β-cell line INS-1, was kindly provided by Dr. Karl Olson at Michigan State University. The cells were grown in RPMI 1640 medium (Invitrogen, Carlsbad, CA, USA) supplemented with 10% fetal bovine serum, 2 mM L-glutamine, 1% streptomycin, gentamycin, amphotericin B antibiotic/antimycotic). Cells were grown at 37°C and 5% CO_2 _atmosphere. 25 cm^2 ^tissue culture treated flasks were used for cell expansion. Upon 80% confluence, cells were detached with trypsin and sub-cultured.

### Oil extractions and analysis

"NOP" oil ("HAIN" brand produced by The Hain Celestial group, Inc, Garden City, NY, USA) and two "HOP" oils ("USDA" kindly provided by United States Department of Agriculture, and "GOLD" produced by Golden Peanut Company, Alpharetta, GA, USA) were used. One mL of both "HOP" oils were extracted twice with chloroform: methanol (1:1; v/v), 95% ethanol and ethanol: ether (2:3; v/v) separately. Extracts were evaporated to dryness under nitrogen and solubilised in 1.0 mL of 5% dimethyl sulfoxide, DMSO, in phosphate buffered saline, PBS.

Fatty acid composition of oils was carried out by J. Leek Associates, Inc. Albany, GA, USA.

### Insulin assay

Secreted insulin was measured using 25 μL of diluted (×10) supernatant from each well (100,000 cells/mL; 200 μL) of 96 well plates using Insulin (Rat) Ultra Sensitive EIA kit from ALPCO diagnostics (Windham, NM, USA). In brief, 25 μl standard, control and unknown were incubated separately with 75 μL conjugate solution, containing insulin antibody, for 120 min at room temperature on a microplate rotator shaking approximately at 800 rpm. Plates were washed 6 times manually and allowed to react with 3, 3', 5, 5'-tetramethylbenzidine peroxidase substrate for at least 15 min at room temperature. Reactions were stopped by addition of 50 μL stop solution. Finally, bi-chromatic absorbance measurement with reference at 650 and 450 nm were performed using a Benchmark Biorad Microplate spectrophotometric reader (BioRad, Sunnyville, CA, USA). Values were expressed either as (mean ± SEM or SD) pg/mL of insulin.

### cAMP assay

cAMP intracellular levels of INS-1 cells were determined using a cAMP Assay Kit obtained from Cayman Chemicals (Ann Arbor, MI, USA) according to manufacturer's instructions. Briefly, INS-1 cells were seeded (2 × 10^5 ^cells/mL) in a 96- well plate in 200 μL medium for 24 hrs and treated with oleic acid at various concentrations for 15 min. Supernatant was removed followed by addition of 100 μL of 0.1 M HCl and 20 minute incubation for cell lysis. Cells were then removed with a rubber policeman, homogenized by repetitive pippetting and centrifuged at 1,000 g. Supernatants were then transferred to a new tube and cAMP levels were measured.

### PPAR-γ binding to PPRE containing oligonucleotides

PPAR-γ binding to peroxisome proliferator responsive element (PPRE) containing oligonucleotides was assayed by measuring binding of nuclear extract homogenates to a specific double stranded DNA oligonucleotide sequence immobilized on a 96 well plate that contains the PPRE. PPAR-γ was detected using a specific primary antibody followed by a secondary antibody conjugated to HRP and colorimetric readout at 450 nm. Nuclear extract preparation was carried out as per manufacturer's instructions (Cayman Chemicals). 3 × 10^6 ^cells were used for each treatment and a total of 2 μg nuclear extract was incubated in each well overnight at 4°C without agitation. Detection antibodies and positive controls were provided with the manufacturer's kit.

### Apoptosis assay

Annexin V/propidium iodide (PI) staining of INS-1 cells cultured with TNF-α and oleic acid at varied concentrations for 24 hrs were washed and adjusted to 1 × 10^6 ^cells/mL in staining buffer. Staining was performed as recommended by BD Pharmingen (San Diego, CA, USA). Cells were analyzed immediately by flow cytometry. Annexin V has high affinity for phosphatidylserine that is translocated to the outer leaflet of the plasma membrane during apoptosis. PI intercalates in the DNA of apoptotic cells with compromised plasma membrane.

### *In vivo *studies

Adult male Kunming (10–12 week old) and KKA^y ^mice (8–10 week old) were supplied by the Institute of Pharmacology, Chinese Academy of Medical Sciences. Mice were given food (different diets according to the need of the experiment) and water *ad libitum*. The animals were kept in a pathogen free environment for a 12-h light, 12-h dark cycle. All animal experiments were conducted according to the guidelines of the local animal use and care committee and executed according to the National Animal Law.

KKA^y ^mice were divided into three groups: a control group (C, kept on normal diet), a Type 2 diabetes group (DM2, kept on a high fat diet), and a peanut oil treated DM2 group (DM2 + peanut oil) kept on a high fat diet. After 7 days of high fat diet when the blood glucose was higher than 15 mM, the mice were administered 0.70 mL/day, ~2.0–2.5% w/w, of peanut oil by gavage for 21 days. On a calorie basis, the high fat diet consisted of 58% fat whereas the normal diet contained 11.4% fat[19].

### Statistics

Statistical significance was established using standard paired student t-test using Sigmaplot^® ^or Microsoft Excel^® ^software.

## Results

### Oleic acid enhances insulin production and reverses the inhibitory effect of TNF-α in INS-1 cells

**Figure 1:**
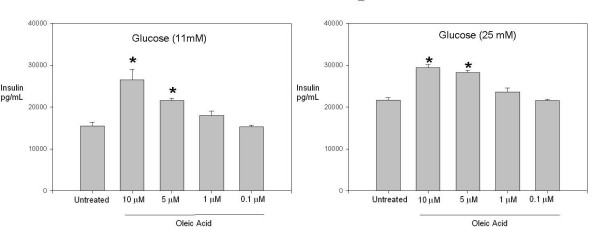
**Insulin production by INS-1 cells treated oleic acid**. (A) INS-1 cells were cultured (11 mM glucose) with varying concentrations of oleic acid for 18–24 hrs at 37°C. Shown is one representative experiment with triplicate values out of three independent experiments. *, p < 0.05 for untreated cells compared to cells treated with 10 μM and 5 μM oleic acid. (B) INS-1 cells were cultured (25 mM glucose) with varying concentrations of oleic acid for 18–24 hrs at 37°C. Shown is one representative experiment with triplicate values out of three independent experiments. *, p < 0.05 for untreated cells compared to cells treated with 10 μM and 5 μM oleic acid.

MUFAs have been shown to exert pleiotropic effects on a variety of cell types. Of particular interest to the scientific community focusing on inflammation, is their ability to modulate insulin production. With this perspective in mind and preliminary findings previously reported, we first assessed the effect of oleic acid on insulin production by the glucose dependent insulin producing rat pancreatic cell line, INS-1[20]. We observed a significant increase in insulin production by INS-1 cells grown in medium containing 11 mM glucose and treated with oleic acid at 10 μM and 5 μM (Fig. 1A). Significant increase in insulin production was observed by INS-1 cells grown in medium containing 25 mM glucose and treated with oleic acid at 10 μM and 5 μM (Fig. 1B).

**Figure 2:**
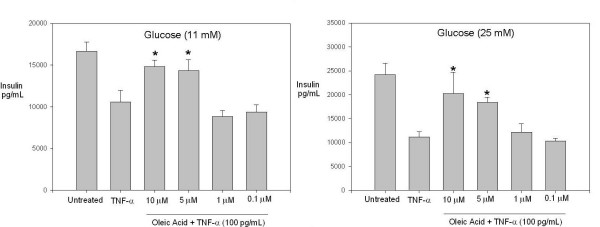
**Increased insulin production by INS-1 cells treated with TNF-α and oleic acid**. (A) INS-1 cells were pre-treated for two hours with varying concentrations of oleic acid in medium containing 11 mM glucose, followed by TNF-α (100 pg/mL) treatment for 18–24 hrs at 37°C. Shown is one representative experiment with triplicate values out of three independent experiments.*, p < 0.05 for cells treated with TNF-α compared to cells treated with TNF-α and 10 μM or 5 μM oleic acid. (B) Cells were pre-treated for two hours with varying concentrations of oleic acid in medium containing 25 mM glucose, followed by TNF-α (100 pg/mL) treatment for 18–24 hrs at 37°C. Shown is one representative experiment with triplicate values out of three independent experiments. *, p < 0.05 for cells treated with TNF-α compared to cells treated with TNF-α and 10 μM or 5 μM oleic acid.

The inflammatory cytokine TNF-α is known to inhibit insulin secretion[5]. Inflammatory cytokines have also been reported to be elevated in both obese individuals and obese individuals that are type II diabetic[21]. Furthermore, obesity and inflammation are considered major risk factors in type II diabetes and hypertension[22]. Besides type II diabetes, chronic inflammation which increases with age, is considered to be a major risk factor for cardiovascular disease[23]. Taking into consideration these findings, we sought to investigate the impact of oleic acid in a simulated inflammatory environment using TNF-α. Oleic acid at 10 μM and 5 μM was able to reverse the inhibitory effect of TNF-α (100 pg/mL) (Fig. 2A). This finding shows that oleic acid is capable of reversing the inhibitory effect of the inflammatory cytokine TNF-α and reveals the potential therapeutic effect of oleic acid and MUFAs in an inflammatory context. We observed a similar effect of insulin production at high glucose (25 mM) culturing conditions with oleic acid treatments at 10 μM and 5 μM and TNF-α (100 pg/mL), (Fig. 2B). Interestingly, these effects are observed after two hours pre-treatment of the cells with oleic acid.

### Effect of oleic acid and TNF-α on apoptosis of INS-1cells

**Figure 3:**
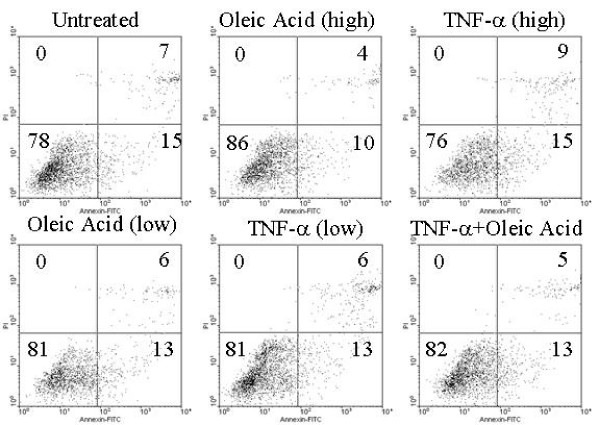
**TNF-α or oleic acid treatment has no apoptotic inducing effect on INS-1 cells**. Cells were pre-treated with low (1 μM) and high (10 μM) oleic acid, low (100 pg/mL) and high (1,000 pg/mL) TNF-α or pre-treated for 2 hours with oleic acid (10 μM) and subsequently treated with TNF-α (100 pg/mL). Twenty-four hours later the apoptotic status of cells was assessed using flow cytometry (Annexin V/PI staining). Results are expressed as percent of apoptotic/necrotic cells (upper right and lower right quadrant). Shown is one representative experiment performed in duplicate out of three independent experiments.

INS-1 cells are susceptible to apoptosis and use of oleic acid and TNF-α has the potential to induce apoptosis. Furthermore, oleic acid could exert its insulinogenic effect by inhibiting apoptosis. To address these possibilities, we assessed the apoptotic status of INS-1 cells under the same experimental conditions that we observed induction of insulin secretion in the presence of oleic acid, as well as reversal of insulin inhibition in the presence of oleic acid and TNF-α. We specifically chose to utilize the annexin V/PI assay primarily for its ability to detect early apoptosis via exposure of phosphatidyl serine on the extracytoplasmic side of the plasma membrane. At 24 hours, we did not observe any statistically significant increase in apoptosis in the presence of high (1,000 pg/mL) or low (100 pg/mL) TNF-α concentration (Fig. 3). We observed a small statistically insignificant reduction in baseline apoptosis in the presence of oleic acid in both high (10 μM) and low (1 μM) concentrations. These findings are consistent with a previous report of the TNF-α inhibitory effect on insulin production from INS-1 cells is not attributable to apoptosis induction[5]. Furthermore, the reversal in insulin secretion inhibition by oleic acid is not related to its anti-apoptotic properties.

### Oleic acid does not induce cAMP elevation in INS-1 cells treated with TNF-α

**Figure 4:**
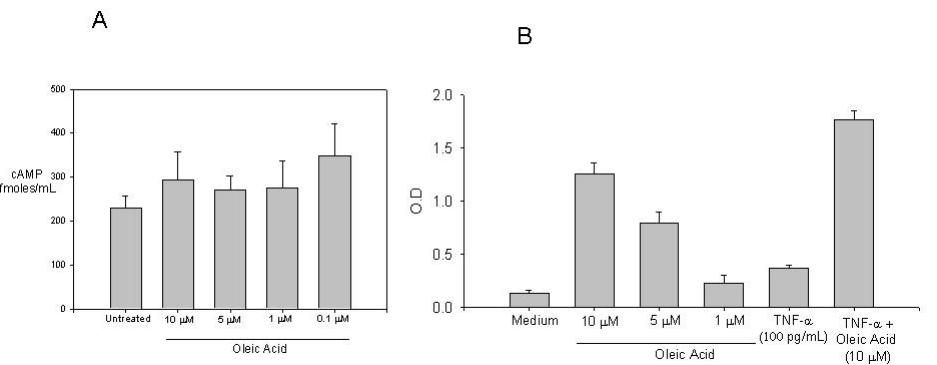
**(A) Oleic acid treatment does not increase intracellular cAMP in INS-1 cells**. Cells were treated with oleic acid at 10 μM, 5 μM, 1 μM and 0.1 μM for 15 min. Cellular homogenates were subjected to cAMP assay. Shown is one representative experiment with triplicate values out of two independent experiments. No significant difference was observed between controls and treatments. **(B) Oleic acid induces translocation of PPAR-γ in INS-1 cells**. Cells were treated with oleic acid at 10 μM, 5 μM, 1 μM, TNF-α (100 pg/mL) or combination of TNF-α and oleic acid (10 μM) for 18–24 hrs. Nuclear extracts were subjected to PPAR-γ detection assay. Shown is one representative experiment with triplicate values out of three independent experiments. *, p < 0.05 for cells treated with oleic acid (10 μM) compared to control and combination of TNF-α (100 pg) with oleic acid (10 μM) compared to cells treated with TNF-α alone.

Fatty acids are capable of binding a number of receptors, both intracellular and extracellular, making the identification of a participating receptor difficult. Recent reports indicate the involvement of the GPR40 receptor in insulin secretion augmentation[24,25]. In an effort to achieve some understanding of the molecular signalling pathway utilized by oleic acid that results in enhancement in insulin secretion in the presence of TNF-α, we measured the intracellular second messenger molecule cyclic AMP after oleic acid treatment. The intracellular cAMP levels in the presence of oleic acid at concentrations ranging from 10 μM, 5 μM, 1 μM and 0.1 μM in the first 15 minutes did not change significantly (Fig. 4A). Furthermore, no significant elevation in cAMP was observed at 30 minutes after oleic acid treatment 10 μM (data not shown).

### Oleic acid induces translocation of PPAR-γ in INS-1 cells

Fatty acids and fatty acid metabolites are known activators of PPAR-γ and have been shown to ameliorate the inflammatory effects of TNF-α [26]. Thiazolidinediones (TZDs), the anti-diabetic class of drugs used extensively for type II diabetes are also PPAR-γ activators[27]. PPAR-γ translocation to the nucleus is thought to be mediating the anti-inflammatory properties of fatty acids. In an effort to elucidate the transcriptional mechanism through which oleic acid augments secretion of insulin in INS-1 cells, we focused on the PPAR-γ receptor, a transcription factor that has the capacity to bind a number of fatty acids and synthetic agonists such as TZDs. The source of PPAR-γ able to bind peroxisome-proliferator responsive elements (PPREs) could be both nuclear and cytosolic. We observed an increase in PPRE binding with oleic acid treatments at 10 μM and 5 μM (Fig. 4B). We also observed a high increase in PPRE binding in co-treatments with oleic acid (10 μM) and TNF-α (100 pg/mL). This is suggestive of the transcriptional role of PPAR-γ in counteracting the inhibitory effects of TNF-α.

### Peanut oil fatty acid analysis

**Figure 5:**
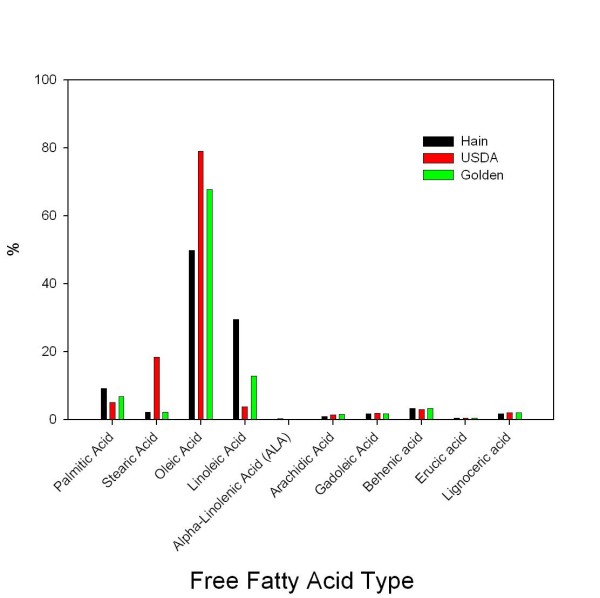
**Fatty acid composition analysis of three peanut oil brands**. One mL from each of the three oils was extracted twice with 1:1 (v/v) chloroform: methanol, 95% ethanol and 2:3 (v/v) ethanol: ether separately. Extracts were evaporated to dryness under nitrogen. The extracts were solubilized in 1.0 mL of 5% DMSO in phosphate buffered saline. Analysis was carried out by J. Leek Associates, Inc. Albany, GA, USA.

We then sought to determine levels of oleic acid in the context of a diet that provides sufficient oleic acid intake that could yield similar anti-diabetic effects such as those observed with purified oleic acid. Oils were extracted from three different brands of peanuts and analyzed for fatty acid composition (J. Leek Associates, Inc. Albany, GA, USA). Although the total mono-unsaturated fatty acid content of the peanut oils was approximately the same, USDA oil contained much higher oleic acid and much less linoleic acid (Fig. 5). Another striking difference is the much higher stearic acid content in USDA oil compared to other samples. Despite the variations in oleic acid content, all three brands of peanut are a good source of oleic acid.

### Effect of peanut oil on blood glucose

**Figure 6:**
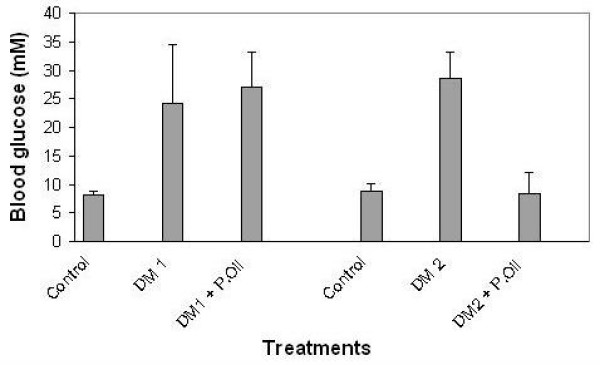
**Robust decrease of blood glucose level in type type 2, but not in type 1 diabetic mice following 21 day peanut oil administration**. Kunming mice were injected intraperitoneally with 40 mg/kg streptozocin to induce type1 diabetes. KKAy mice were maintained on a high fat diet for 10 days to induce type 2 diabetes. Once the mice were characterized to be diabetic, either type I or type II, 0.70 mL of peanut oil was administered by gavages to those mice for 21 days. Control mice were maintained on a standard diet and all other mice were maintained on a high fat diet. At the end of the experiments the fasting blood glucose levels were measured and expressed as mean ± SD (mM glucose) (p = 0.0004 between DM 2 and DM 2 + P. Oil; p = 0.0003 between control and DM 2 and p = 0.05 between control and DM 1).

Based on the fatty acid analysis of the three peanut brands tested, which revealed a high oleic acid content in all three, we assessed the impact of peanut oil consumption on fasting blood glucose levels in experimentally induced type I and II diabetic mice. Once the blood glucose levels reached 15.0 mM or higher, 0.70 mL peanut oil was administered daily for three weeks. A dosage level of peanut oil for the mice was chosen based upon studies using dietary oils in pigs supplemented to 40 g olive or sunflower oil per kg body weight[28]. Our exploratory study using mice employed 0.70 mL peanut oil, or approximately 18 g oil per kg body weight per day for three weeks. Clinical studies demonstrating beneficial effects of peanut butter were found with a dietary intake of >20 g/day based upon a dietary questionnaire with data followed up to 16 years; using an average body weight of 65 kg, this corresponds to a dosage of approximately 0.3 g peanut butter/kg body weight per day. The blood glucose levels in all type 2 (n = 8) diabetic mice, that received peanut oil, was significantly reduced (8.3 ± 3.7; p = 0.0004 between DM2 and DM2 + peanut oil; see Fig. 6) whereas no significant changes of blood glucose (27 ± 6) were observed for type 1 (n = 5) diabetic mice that received peanut oil. Fasting blood glucose levels of both type 1 diabetic (29 ± 4; n = 4) and type 2 diabetic (24 ± 10; n = 7) mice that did not receive peanut oil remained elevated at the end of the 21 day treatment.

## Discussion

The present study reveals the anti-diabetic and anti-inflammatory properties of oleic acid. Oleic acid increases insulin secretion in the glucose sensitive INS-1 cell line. Even more noteworthy however, is the ability of oleic acid to increase insulin secretion in the presence of the inflammatory cytokine TNF-α. Previous studies have implicated TNF-α plasma levels with type II diabetes[29]. The direct implication of TNF-α levels in diabetes points to the key role inflammation plays in pathogenesis. Oleic acid treatment was recently shown to influence expression of approximately 14 genes. These include transcription factors such as the early response growth factor 1 (EGR1) and ubiquitin, a protein involved in proteololysis [30].

Fatty acids have been shown to be involved in both pro-apoptotic and anti-apoptotic pathways[31,32]. Despite the relatively short duration of oleic acid treatment both in the presence and absence of TNF-α, we considered the possibility that the reversal in inhibition was attributable to an anti-apoptotic effect. Utilizing annexin V and PI, an assay with an ability to detect apoptosis at the very early stages of its occurrence, we did not see any appreciable differences when compared to controls. The possibility of oleic acid influencing apoptosis in the long term exists, however our study focused on the short-term impact.

The specific molecular mechanisms via which unsaturated fatty acids exert their influence, including oleic acid, are quite varied. Our findings show that the PPAR-γ receptor may be involved in the molecular mechanism that reverses TNF-α insulin inhibition. The detection of PPAR-γ via interaction with PPREs is suggestive of an increased availability of this transcription factor at the nuclear level. The specific promoters or regulatory sequences that are influenced by the presence PPAR-γ due to oleic acid treatment are not addressed in the present study, but given the observed outcome, it is evident that it is reversing the negative impact of TNF-α signalling.

The *in vivo *system we utilized is based on the KKA^y ^type II diabetic mouse, which is characterized by high levels of TNF-α [33]. In this study, consumption of peanut oil, which is high in oleic acid, was able to reverse the high glucose levels of all type 2 diabetic mice (8 out of 8) and normalize values by 21 day treatment of peanut oil, whereas the blood glucose levels of type 1 diabetic mice remained unaffected. These findings are consistent with previous studies that have shown that long term consumption of peanuts and peanut butter lowered the risk of type 2 diabetes in women[34].

Peanuts are part of the legume family and are rich in healthy types of fat, and some proteins, fiber, magnesium, antioxidant vitamin (vitamin E), phytolaxin and resveratrol. Both antioxidant and resveratrol could also contribute to the beneficial effects of peanuts. Resveratrol, one of the compounds in grapes/red wine thought to be responsible for health benefits, is also present in peanuts but at lower levels (red wine averages 160 μg of resveratrol/fluid ounce, compared to peanuts, which average 73 μg per ounce[35]. In general, peanuts contain various proteins ranging from 14 kDa to 90 kDa. Among them 62.5 kDa maturity associated protein, water soluble glycosylated polypeptides (32.8 kDa and 34.8 kDa) and some lipoprotein rich in oleic and linoleic acids are the major protein components [36-38]. However, based on oil extractions we carried out and tested, the ingredient responsible for the observed effect is very likely oleic acid. The peanut oil analysis shows the striking prevalence of oleic acid in all brands tested.

The widespread practice of hydrogenation of fatty acids used in processed foods in the western world including United States and parts of Europe, is considered to be a risk factor for a number of diseases. A number of studies have shown the unique properties of unsaturated fatty acids and the importance of maintaining the intake of unsaturated fatty acid as high as possible [39-41]. In a comprehensive metabolic study Segall *et al*, showed that chronic exposure of INS -1 cells, to oleate produced an increased oxidation and esterification of fatty acids that might contribute to high basal insulin secretion via "increased production of reducing equivalents and/or the generation of complex lipid messenger molecule(s)"[42]. The mechanisms of the anti-diabetogenic action of oleic acid are very likely multi-faceted. The role of oleic acid in the presence of the proinflammatory cytokine TNF-α is clearly beneficial in this study. Dietary supplementation with MUFAs and PUFAs has been reported to inhibit other inflammatory cytokines, such as IL-1 and IL-12 [43,44]. Dietary fatty acid intake, particularly unsaturated fatty acids, may also alter the composition of membrane phospholipids in addition to binding nuclear transcription factors. Changes in phospholipid composition may in turn change membrane fluidity thereby altering the binding of cytokines to their corresponding receptors.

## Conclusion

In this study, oleic acid has been shown to increase insulin production and to reverse the inhibitory insulin effect of TNF-α. Peanut oil, high in oleic acid, was able to ameliorate the diabetic symptoms of type II diabetic mice characterized by inflammation. The findings are consistent with earlier clinical and epidemiological findings that peanuts are beneficial in lowering the risk of type 2 diabetes in women. This study provides further evidence to the importance of regulating inflammation using naturally occurring molecules found in certain foods. Furthermore, it indicates the importance of unsaturated fatty acids and their potential to regulate inflammation.

## Abbreviations

"NOP": Normal oleic acid peanut; "HOP": high oleic acid peanut; MUFA: mono-unsaturated fatty acids; PUFA: poly-unsaturated fatty acids; GLP-1: glucagon like peptide7–36; HDL: high density lipoprotein; LDL: low density lipoprotein; CD: Crohn's Disease; RA: Rheumatoid Arthritis; hs-CRP: High sensitivity C-Reactive Protein; TZDs: Thiazolidinediones; TNF-α: Tumor Necrosis Factor-alpha; PPAR-γ: Peroxisome proliferator activated receptor-gamma; PPRE: Peroxisome proliferator responsive elements.

## Competing interests

The authors declare that they have no competing interests.

## Authors' contributions

EKV and JT had substantial contributions to conception, design, interpretation of data and writing the manuscript. EKV, JT, AG, CG and GC carried out immunological and in vivo assays. All authors read and approved the final manuscript.

## References

[B1] Cavaillon JM (1995). Cytokines in inflammation. C R Seances Soc Biol Fil.

[B2] Vassiliou E, Jing H, Ganea D (2003). Prostaglandin E2 inhibits TNF production in murine bone marrow-derived dendritic cells. Cell Immunol.

[B3] Yudkin JS (2007). Inflammation, obesity, and the metabolic syndrome. Horm Metab Res.

[B4] Kim HE, Choi SE, Lee SJ, Lee JH, Lee YJ, Kang SS, Chun J, Kang Y (2008). Tumour necrosis factor-alpha-induced glucose-stimulated insulin secretion inhibition in INS-1 cells is ascribed to a reduction of the glucose-stimulated Ca2+ influx. J Endocrinol.

[B5] Zhang S, Kim KH (1995). TNF-alpha inhibits glucose-induced insulin secretion in a pancreatic beta-cell line (INS-1). FEBS Lett.

[B6] del Aguila LF, Claffey KP, Kirwan JP (1999). TNF-alpha impairs insulin signaling and insulin stimulation of glucose uptake in C2C12 muscle cells. Am J Physiol.

[B7] Ridker PM, Danielson E, Fonseca FA, Genest J, Gotto AM, Kastelein JJ, Koenig W, Libby P, Lorenzatti HA, MacFadyen JG, Nordestgaard BG, Shepherd J, Willerson JT, Glynn RJ (2008). Rosuvastatin to prevent vascular events in men and women with elevated C-reactive protein. N Engl J Med.

[B8] Jimenez-Gomez Y, Lopez-Miranda J, Blanco-Colio LM, Marin C, Perez-Martinez P, Ruano J, Paniagua JA, Rodriguez F, Egido J, Perez-Jimerez F (2008). Olive oil and walnut breakfasts reduce the postprandial inflammatory response in mononuclear cells compared with a butter breakfast in healthy men. Atherosclerosis.

[B9] Micallef MA, Garg ML (2009). Anti-inflammatory and cardioprotective effects of n-3 polyunsaturated fatty acids and plant sterols in hyperlipidemic individuals. Atherosclerosis.

[B10] Ouellet V, Weisnagel SJ, Marois J, Bergeron J, Julien P, Gougeon R, Tchernof A, Holub BJ, Jacques H (2008). Dietary cod protein reduces plasma C-reactive protein in insulin-resistant men and women. J Nutr.

[B11] Lichtenstein AH, Ausman LM, Carrasco W, Jenner JL, Ordovas JM, Schaefer EJ (1993). Hydrogenation impairs the hypolipidemic effect of corn oil in humans. Hydrogenation, trans fatty acids, and plasma lipids. Arterioscler Thromb.

[B12] Vassiliou EK, Kesler OM, Tadros JH, Ganea D (2008). Bone marrow-derived dendritic cells generated in the presence of resolvin E1 induce apoptosis of activated CD4+ T cells. J Immunol.

[B13] Arita M, Yoshida M, Hong S, Tjonahen E, Glickman JN, Petasis NA, Blumberg RS, Serhan CN (2005). Resolvin E1, an endogenous lipid mediator derived from omega-3 eicosapentaenoic acid, protects against 2,4,6-trinitrobenzene sulfonic acid-induced colitis. Proc Natl Acad Sci USA.

[B14] Li H, Ruan XZ, Powis SH, Fernando R, Mon WY, Wheeler DC, Moorhead JF, Varghese Z (2005). EPA and DHA reduce LPS-induced inflammation responses in HK-2 cells: evidence for a PPAR-gamma-dependent mechanism. Kidney Int.

[B15] Trayhurn P, Wood IS (2004). Adipokines: inflammation and the pleiotropic role of white adipose tissue. Br J Nutr.

[B16] Tsimikas S, Philis-Tsimikas A, Alexopoulos S, Sigari F, Lee C, Reaven PD (1999). LDL isolated from Greek subjects on a typical diet or from American subjects on an oleate-supplemented diet induces less monocyte chemotaxis and adhesion when exposed to oxidative stress. Arterioscler Thromb Vasc Biol.

[B17] Jossa F, Mancini M (1996). The Mediterranean diet in the prevention of arteriosclerosis. Recenti Prog Med.

[B18] Panagiotakos DB, Dimakopoulou K, Katsouyanni K, Bellander T, Grau M, Koenig W, Lanki T, Pistelli R, Schneider A, Peters A (2009). Mediterranean diet and inflammatory response in myocardial infarction survivors. Int J Epidemiol.

[B19] Winzell MS, Ahren B (2004). The high-fat diet-fed mouse: a model for studying mechanisms and treatment of impaired glucose tolerance and type 2 diabetes. Diabetes.

[B20] Chakraborty G, Zhao YM, Sheng SL, Zhao W, Li LH, Ruskin MS, Toney JH (2005). Peanut oil lowers blood glucose in models of type II but not type I diabetic mice. ADA.

[B21] Catalan V, Gomez-Ambrosi J, Ramirez B, Rotellar F, Pastor C, Silva C, Rotellar F, Gil MJ, Cienfuegos JA, Salvador J, Vendrell J, Fruhbeck G (2007). Proinflammatory cytokines in obesity: impact of type 2 diabetes mellitus and gastric bypass. Obes Surg.

[B22] Pitsavos C, Chrysohoou C, Panagiotakos DB, Lentzas Y, Stefanadis C (2008). Abdominal obesity and inflammation predicts hypertension among prehypertensive men and women: the ATTICA Study. Heart Vessels.

[B23] Kalofoutis C, Piperi C, Zisaki A, Singh J, Harris F, Phoenix D, Alaveras A, Kalofoutis A (2006). Differences in expression of cardiovascular risk factors among type 2 diabetes mellitus patients of different age. Ann N Y Acad Sci.

[B24] Kebede M, Alquier T, Latour MG, Semache M, Tremblay C, Poitout V (2008). The fatty acid receptor GPR40 plays a role in insulin secretion in vivo after high-fat feeding. Diabetes.

[B25] Nagasumi K, Esaki R, Iwachidow K, Yasuhara Y, Ogi K, Tanaka H, Nakata M, Yano T, Shimakawa K, Taketomi S, Takeuchi K, Odaka H, Kaisho Y (2009). Overexpression of GPR40 in Pancreatic {beta}-Cells Augments Glucose Stimulated Insulin Secretion and Improves Glucose Tolerance in Normal and Diabetic Mice. Diabetes.

[B26] Calabro P, Samudio I, Safe SH, Willerson JT, Yeh ET (2005). Inhibition of tumor-necrosis-factor-alpha induced endothelial cell activation by a new class of PPAR-gamma agonists. An in vitro study showing receptor-independent effects. J Vasc Res.

[B27] Sood V, Colleran K, Burge MR (2000). Thiazolidinediones: a comparative review of approved uses. Diabetes Technol Ther.

[B28] Navarro MA, Acin S, Carnicer R, Guzman-Garcia MA, Arbones-Mainar JM, Surra JC, Cebrian JA, Arnal C, Isabel B, Lopez-Bote CJ, Osada J (2004). Response of ApoA-IV in pigs to long-term increased dietary oil intake and to the degree of unsaturation of the fatty acids. Br J Nutr.

[B29] Plomgaard P, Nielsen AR, Fischer CP, Mortensen OH, Broholm C, Penkowa M, Krogh-Madsen R, Erikstrup C, Petersen AM, Lindegaard B, Taudorf S, Pedersen BK (2007). Associations between insulin resistance and TNF-alpha in plasma, skeletal muscle and adipose tissue in humans with and without type 2 diabetes. Diabetologia.

[B30] Vock C, Gleissner M, Klapper M, Doring F (2008). Oleate regulates genes controlled by signaling pathways of mitogen-activated protein kinase, insulin, and hypoxia. Nutr Res.

[B31] Koshkin V, Dai FF, Robson-Doucette CA, Chan CB, Wheeler MB (2008). Limited mitochondrial permeabilization is an early manifestation of palmitate-induced lipotoxicity in pancreatic beta-cells. J Biol Chem.

[B32] Guo W, Wong S, Xie W, Lei T, Luo Z (2007). Palmitate modulates intracellular signaling, induces endoplasmic reticulum stress, and causes apoptosis in mouse 3T3-L1 and rat primary preadipocytes. Am J Physiol Endocrinol Metab.

[B33] Fu L, Isobe K, Zeng Q, Suzukawa K, Takekoshi K, Kawakami Y (2007). beta-adrenoceptor agonists downregulate adiponectin, but upregulate adiponectin receptor 2 and tumor necrosis factor-alpha expression in adipocytes. Eur J Pharmacol.

[B34] Jiang R, Manson JE, Stampfer MJ, Liu S, Willett WC, Hu FB (2002). Nut and peanut butter consumption and risk of type 2 diabetes in women. JAMA.

[B35] Sanders TH, McMichael RW, Hendrix KW (2000). Occurrence of resveratrol in edible peanuts. J Agric Food Chem.

[B36] Alam M, Basha SM, Boyd LC (2000). Characterization of methanol-soluble and methanol-insoluble proteins from developing peanut seed. J Agric Food Chem.

[B37] Sathe SK, Hamaker BR, Sze-Tao KW, Venkatachalam M (2002). Isolation, purification, and biochemical characterization of a novel water soluble protein from Inca peanut (Plukenetia volubilis L.). J Agric Food Chem.

[B38] Bland JM, Lax AR (2000). Isolation and characterization of a peanut maturity-associated protein. J Agric Food Chem.

[B39] Bersamin A, Luick BR, King IB, Stern JS, Zidenberg-Cherr S (2008). Westernizing diets influence fat intake, red blood cell fatty acid composition, and health in remote Alaskan Native communities in the center for Alaska Native health study. J Am Diet Assoc.

[B40] Lardinois CK (1987). The role of omega 3 fatty acids on insulin secretion and insulin sensitivity. Med Hypotheses.

[B41] Mulvad G, Pedersen HS, Hansen JC, Dewailly E, Jul E, Pedersen M, Deguchi Y, Newman WP, Malcom GT, Tracy RE, Middaugh JP, Bjerreggard P (1996). The Inuit diet. Fatty acids and antioxidants, their role in ischemic heart disease, and exposure to organochlorines and heavy metals. An international study. Arctic Med Res.

[B42] Segall L, Lameloise N, Assimacopoulos-Jeannet F, Roche E, Corkey P, Thumelin S, Corkey BE, Prentki M (1999). Lipid rather than glucose metabolism is implicated in altered insulin secretion caused by oleate in INS-1 cells. Am J Physiol.

[B43] Grimble RF, Tappia PS (1998). Modulation of pro-inflammatory cytokine biology by unsaturated fatty acids. Z Ernahrungswiss.

[B44] Zhang M, Fritsche KL (2004). Fatty acid-mediated inhibition of IL-12 production by murine macrophages is independent of PPARgamma. Br J Nutr.

